# SOT at 50: A Proud Legacy, A Vibrant Future

**DOI:** 10.1289/ehp.1103511

**Published:** 2011-03

**Authors:** Linda S. Birnbaum

**Affiliations:** Director, NIEHS and NTP, National Institutes of Health, Department of Health and Human Services, Research Triangle Park, North Carolina, E-mail: birnbaumls@niehs.nih.gov

The National Institute of Environmental Health Sciences (NIEHS) joins with the entire environmental health sciences community in celebrating the 50th anniversary of the Society of Toxicology (SOT). This issue of *Environmental Health Perspectives* (*EHP*) contains several articles in the field of toxicology and includes the poster *Benchmarks in Toxicology*, which delineates the most significant accomplishments in the field, from Phillipus Aurolus Paracelsus, the sixteenth-century scientist considered to be the “father of toxicology,” to today’s cutting-edge technologies and initiatives that are enabling the current radical transformation of the discipline. *EHP’*s March podcast features an interview with Peter Goering, a research toxicologist at the U.S. Food and Drug Administration and secretary of the Society of Toxicology. In the interview, he discusses how the benchmarks were selected and provides more details about specific benchmarks.

The SOT was founded on 6 March 1961 by a visionary group of nine founders who were joined by 183 charter members. Today the SOT is a global organization with > 6,500 members from all over the world. It is a broad tent, with everyone in the field welcomed and encouraged to participate, including basic scientists, regulators, academics, industry professionals, government officials, consultants, and representatives from non-governmental organizations. With such a richly diverse membership, many different viewpoints from many different types of scientists are heard, which makes for a robust, healthy scientific dialogue. The SOT’s strong emphasis on mentoring and education programs, along with its recruitment of younger members into interesting roles and committee assignments in the organization, helps to ensure commitment to the field’s health by the next generation of toxicologists.

The SOT has long been intensely devoted to building for the future of toxicology and the professional development of its membership. For example, the SOT currently has 26 specialty sections focusing on specific areas of toxicology. These science-based programs are complemented by 27 elected and appointed committees, subcommittees, and task forces; 18 regional chapters; and 6 Special Interest Groups (SIGs). The SIGs, which include the American Association of Chinese in Toxicology, the Association of Scientists of Indian Origin, the Hispanic Organization of Toxicologists, the Korean Toxicologists Association in America, Toxicologists of African Origin, and Women in Toxicology, promote the diversity and inclusiveness of the organization by recruiting toxicologists who share a common interest in toxicological research issues germane to their cultures and communities. The SOT also maintains a global perspective, playing a major role in the International Union of Toxicology (IUTOX) as one of the member societies on the IUTOX executive committee.

Over the past 50 years the SOT has played a key role in advancing the science of toxicology as it has evolved from a science devoted to studying poisons and adverse effects of chemical exposures to a science devoted to studying safety. As toxicology has changed from a descriptive, observational discipline to one with an emphasis on predictive power and protection of public health, the SOT has been at the forefront of this profound reorientation.

I first joined the SOT in 1982. Since then I have had the honor of serving as the society’s president (2004–2005) and in several other leadership roles. So it should come as no surprise that the SOT is near and dear to my heart, and I can confidently state that it is an asset to of all of us at the NIEHS and the National Toxicology Program (NTP).

For me, the SOT Annual Meeting has always been an essential conference to attend to hear about the newest findings and newest areas of research in toxicology. I’ve always enjoyed the plenary sessions, the special lectures, such as the Medical Research Council keynote lectures, and the many symposia focusing on new information in various areas. I am always inspired by the workshops and roundtables that take place at the meeting; the formats of those sessions facilitate positive and open discussion between people with differing views in a nonthreatening atmosphere.

The quality of the science presented at the meeting is extremely high. I would especially encourage younger scientists in the field to attend and make the most of their experience. There are numerous opportunities for junior researchers to meet and interact with more senior scientists, particularly at the poster sessions and mentoring programs. As I can testify from personal experience, those interactions can have long-lasting benefits to your scientific and career success.

I am proud that the NIEHS and NTP have been a major presence at the SOT Annual Meeting and ToxExpo in recent years. In 2010, we hosted > 30 sessions and presented > 60 posters. We also inaugurated an innovative service called SOT Live Update, which provided nearly 100 up-to-the-minute observations about conference events and activities online and on Twitter. And of course, we had a large booth at the ToxExpo where conference goers could pick up the latest issue of *EHP.*

At this year’s 50th Annual Meeting, 6–10 March in Washington, DC, we will once again lead the proceedings with investigators and program officers presenting many posters and sessions, including sessions to answer questions about applying for grants. We will host an exhibit booth and provide live online updates by conference participants (including me), which will be continuously posted on the NIEHS website (http://www.niehs.nih.gov) and on Twitter (http://twitter.com/NIEHS). That way, those of you who cannot attend will still be able to get the flavor of this most important event on the toxicology calendar.

This year’s meeting is especially significant, of course. With a variety of terrific activities planned to fete the SOT’s 50th anniversary, it will literally be a once-in-a-lifetime experience. I can’t wait to join our friends and colleagues as we gather to recognize 50 remarkable years of SOT. It will be a great opportunity to fondly celebrate the past as we eagerly anticipate the future.

See you, I hope, in DC!

## Figures and Tables

**Figure:**
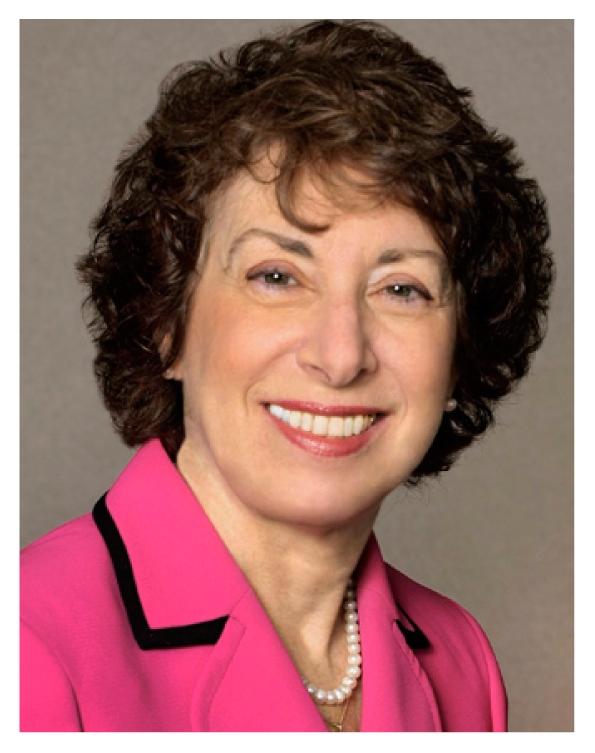
Linda S. Birnbaum

